# An Unusual Presentation of Bladder Carcinoma in a Visceral Hernia: A Case Report and Literature Review

**DOI:** 10.1002/cnr2.70128

**Published:** 2025-02-02

**Authors:** Pouya Ebrahimi, Abbas Mahdavian, Maryam Mousavinejad, Delaram J. Ghadimi, Maryam Taheri, Fatemeh Mahmudi

**Affiliations:** ^1^ Tehran Heart Center Cardiovascular Disease Research Institute, Tehran University of Medical Sciences Tehran Iran; ^2^ Ahvaz Jundishapur University of Medical Sciences Ahvaz Iran; ^3^ Cancer Research Center Ahvaz Jundishapur University of Medical Sciences Ahvaz Iran; ^4^ School of Medicine, Shahid Beheshti University of Medical Sciences Tehran Iran; ^5^ Department of Pathology, School of Medicine Hamadan University of Medical Sciences Hamadan Iran; ^6^ Department of Pathology, School of Medicine Isfahan University of Medical Sciences Isfahan Iran

**Keywords:** abdominal hernia, bladder carcinoma, cancer, case report, urogenital system

## Abstract

**Introduction:**

Bladder carcinoma (BC) is the most prevalent malignancy of the urinary system. These cancers are primarily seen in adults > 60 years old and mostly present with microscopic or frank hematuria or obstruction of the urinary system. However, these rare cancers can be found in hernias.

**Case Presentation:**

This report discusses a rare, localized bladder urothelial carcinoma (UC) manifestation. The patient had presented with lower abdominal pain several times. However, no accurate diagnosis was made due to the unspecified pain features. After being referred to a radiologic evaluation with ultrasonography, a bladder hernia was detected entering the abdominal wall, and it contained an unusual mass. Further evaluations revealed the malignant feature of the tumor. The abdominal wall hernia was replaced, and a TURP procedure was performed. The resulting sample showed UC without the involvement of the muscle layer.

**Conclusion:**

One of the most common malignancies of the urogenital and reproductive systems in male patients is BCs. They are most commonly seen in men older than 60 years old with a history of smoking. The prevalent manifestations of cancer are microscopic or macroscopic hematuria, urinary obstruction, and abdominal pain. A rare but previously reported bladder cancer location is within inguinal or abdominal hernias. The diagnosis of this cancer is not always straightforward, and delays can result in the spread of malignancy and the transition of the patient's clinical condition to a poorer prognosis.

**Clinical Key Message:**

The presentation of bladder cancer is not always accompanied by typical symptoms such as hematuria or urinary obstruction. Patients with persistent lower abdominal pain should be evaluated to rule out bladder malignancy. These tumors might be hidden within abdominal or inguinal hernias, and more radiologic accuracy is demanded for their diagnosis.

## 1 Introduction

Urogenital and reproductive system cancers are some of the most prevalent malignancies seen in men > 60 years old [1, 2]. The most common cancer of the urinary system is bladder carcinoma (BC), mainly seen in the form of urothelial carcinoma (UC), seen in 90% of BC cases and representing the fourth most common cancer in the United States (US) in patients older than 80 [3, 4]. There is a considerable difference between genders in the prevalence of UC, with almost 75% of bladder cancer incidences occurring in men [5]. Although the prevalence of UC is higher in white men, female patients are more prevalently diagnosed with advanced disease, and this is partly explained by later evaluation of hematuria in women [5]. There is no consensus on the factors influencing the incidence of these types of cancers. However, smoking and exposure to chemicals such as arsenic and aromatic amines have been studied and revealed to be associated with a higher incidence of UC. One of the mechanisms of the effect of these factors seems to be through the changes in the genetic factors of the bladder urothelial cells [6, 7]. Moreover, aging, male gender, unhealthy diets, and obesity have been shown to be contributing factors to urothelial cancers [8]. Interestingly, cardiovascular diseases have been found to be protective factors against bladder cancers [9]. Smoking cessation has also been reported to be a protective factor for urothelial cancer [9].

The most common presentation of bladder cancer is either microscopic or frank hematuria. However, irritative symptoms of the urinary tract, as well as suprapubic and abdominal pain, are mainly caused by the obstruction of the ureter or urethra, which are other common presentations [10, 11]. On the other hand, some of these tumors are diagnosed as an incidental finding, which is defined as the diagnosis of cancer during evaluation for a reason other than BC [12]. However, in rare cases, the diagnosis of BC is challenged by the location of the mass. A small number of studies have reported the diagnosis of BC during the evaluation of various types of hernias [13]. The presence of a vesical hernia is a relatively rare condition, and the coexistence of BC has been reported very rarely [14]. Most of these reports described the emergence of tumors in the inguinal or scrotal hernia. Kandemirli et al. described the diagnosis of BC in a visceral hernia entering the right inguinal canal [15]. Other bladder tumors in hernias have also been found entering other parts, such as the scrotum or inguinal canals [16].

This study presents a case of a bladder tumor located in a visceral bladder hernia, found during the evaluation of an older man with abdominal and flank pain. To our knowledge, this is the first case presented with a malignant tumor in the visceral vesical hernia.

## 2 Case Presentation

### 2.1 Patient's History and Examination

A 73‐year‐old male came to our tertiary outpatient urology clinic (Omid Hospital in Lali) with complaints of on‐and‐off sharp right flank and lower abdominal pain that began 2 months before his presentation (22 September 2023). He made several visits seeking relief from his pain, but none of them were effective, and the pain was not responsive to the recommended treatments. The patient had a history of smoking for almost 50 years in the practice of smoking several cigarettes in succession. There was no history of trauma or other systemic symptoms. The pain was not responsive to non‐steroidal anti‐inflammatory drugs (NSAIDs) and other painkillers. The patient's general appearance and vital signs were unremarkable. Physical examination revealed mild lower abdominal tenderness and bulging in the left inguinal area, which was more prominent while the patient was standing and more apparent when the patient was asked to cough or use the Valsalva maneuver. There was no other remarkable finding in the physical exam.

### 2.2 Methods

**FIGURE 1:**
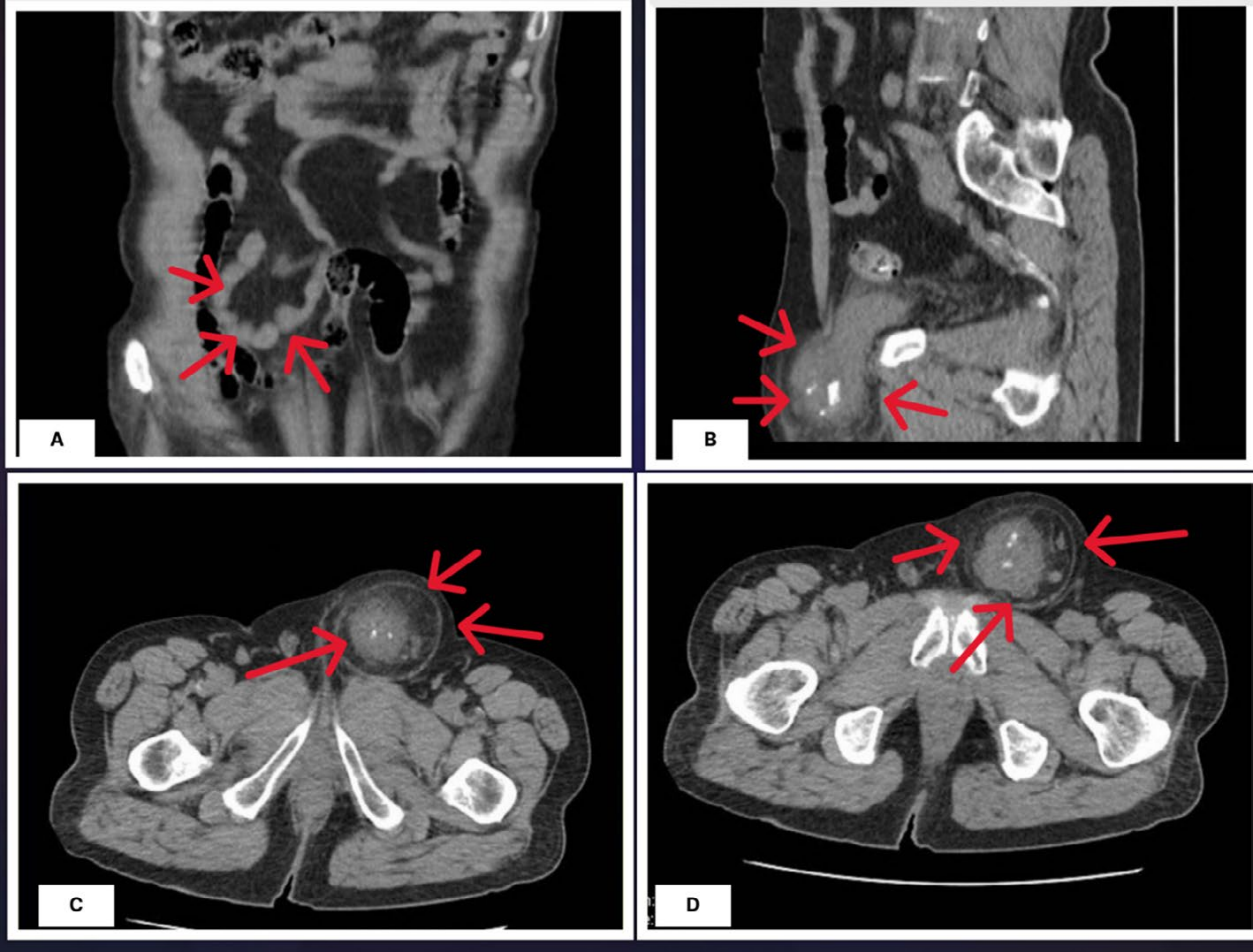
Abdominal and pelvic CT scan (with IV and oral contrast) showed a calcified, irregularly round mass (red arrows) herniating into the abdominal wall, suggesting the probability of a malignant tumor (A and B: Sagittal view, C and D: Axial view).

Considering the unresolved pain, imaging of the abdomen and pelvis was considered the first step of the radiologic evaluation. Ultrasonography of the left inguinal, with a 5–12 MHz device and performed by an expert radiologist, demonstrated a palpable mass with fat stranding in the hypoderm tissue. There was also a hypoecho and heterogeneous mass (52*54 mm), which was aligned with the left anterior wall of the bladder. The mass extended into the left side of the urinary bladder through a 21‐mm defect. The central part of the mass was prominently seen due to several calcifications, with a total diameter of 34 mm. The bladder's left wall was thickened and uneven, with a thickness of roughly 16 mm. The provisional diagnosis suggested by the radiologist was a left bladder wall diverticulum herniated into the abdominal wall, containing probable transitional cell carcinoma (TCC) or adenocarcinoma of the bladder. Considering the high probability of a malignant diagnosis, an abdominal and pelvic computed tomography (CT) scan with intravenous (IV) and oral contrast was done on 24 September 2023 that confirmed the presence of a herniated part of the bladder with a calcified mass, suggesting the malignancy of the bladder herniated into the abdominal wall, as depicted in Figure 1. There was no evidence suggesting the metastasis of the tumor in the abdominal or pelvic cavity. Laboratory data revealed no abnormalities except for microscopic hematuria in the urinalysis (Table 1).

**TABLE 1:** Laboratory findings of the patient.

Test	Result	Reference range
Red blood cell count (RBC) (10^6^/μL)	5.2	4.2–5.5
Hemoglobin (gr/dL)	14.7	12–16
White blood cell count (WBC) (per μL)	6310	4000–11 000
Mean corpuscular volume (MVC) (fL)	88.8	80–99
Hematocrit (%)	45.1	37–47
Platelet (per μL)	165 000	150 000–400 000
Neutrophils (%)	75%	40–75
Lymphocytes (%)	20.7%	20–45
Eosinophils (%)	4.3%	0–6
Lipid profile, coagulation factors, and troponin		
Cholesterol	165	Up to 200
Triglyceride (mg/dL)	70 mg/dL	Up to 150
Low density lipid (mg/dL)	96 mg/dL	Up to 130
High density lipid (mg/dL)	48 mg/dL	> 45 mg/dL
Blood sugar (mg/dL)	99 mg/dL	74–106 mg/dL
Lactate dehydrogenase (mg/dL)	293 Iu/L	235–470 Iu/L
Potassium (K^+^) (meq/L)	4.5	3.5–5.3 meq/L
Creatinine (mg/dL)	1.10	0.5–1.00
Urea (mg/dL)	32	13–43
Prothrombin time (PT)	12.3 s	11–13 s
Partial thromboplastin time (PTT)	27 s	25–38 s
International normalized ratio	1.25	1–1.5

*Note:* Urine analysis: microscopic hematuria (RBC: 9–10) was detected.

**FIGURE 2:**
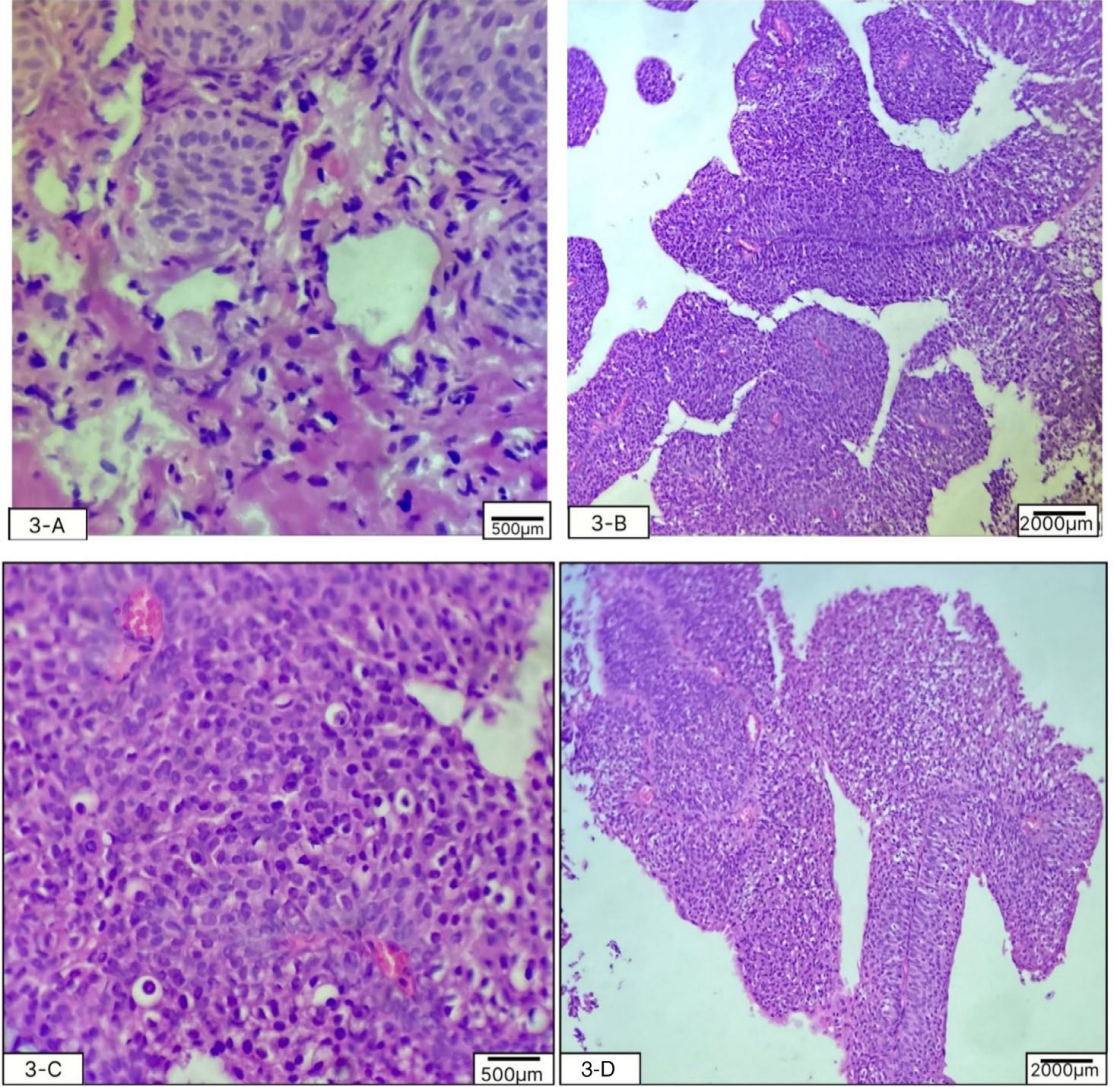
Histopathologic evaluation showed irregular nests of small round‐to‐oval urothelial cells with bland cytology, along with intact inner and outer muscle fibers of the muscularis propria and the adventitia (magnification: A and C: ×40, B and D: ×10).

The patient was admitted for cystoscopy and biopsy of the tumor. The cystoscopy, performed by an expert urologist on 26 September 2023, showed a herniated, irregularly round, calcified mass with an approximate diameter of 50 mm, herniated through the abdominal wall. The biopsy was performed with the aim of detailed evaluation. After the tumor surface was irrigated with an irrigation channel, seven samples were obtained from the trigone, bladder dome, and the right, left, anterior, and posterior bladder walls. This was done to map the bladder mass using a 3‐F cold cup forceps (Piranha). Then, the biopsy device and ureteroscope were removed together to avoid shearing and loss of the sample. Then, aspiration for cytology was repeated to ensure a sufficient sample was taken and sent for pathologic evaluation. Histopathological evaluation of the samples with optical microscopy, Hematoxylin and Eosin staining, and ×100 magnification showed features consistent with urothelial cell carcinoma without invasion of the bladder muscles (1st October 2023). High‐power histopathology evaluation showed irregular nests of small, round‐to‐oval urothelial cells with bland cytology (low grade). The inner and outer muscle fibers of the muscularis propria and adventitia were intact (Figure 2). No evidence of lymphovascular invasion was noted (Table 2). The carcinoma in situ component was not present in the biopsy specimen. The stage of the tumor, considering the involvement, was seen in both imaging (pelvic CT scan) and histopathological evaluation (muscle invasion without the involvement of adventitia). The medical team discussed the diagnosis, therapeutic options, and the need to perform a positron emission tomography (PET) scan for further evaluation with the patient and his family.

**TABLE 2:** Urothelial cell carcinoma diagnostic features [17, 18, 19].

Indicator	Results
Pathological tumor grade	Low‐grade, small round‐to‐oval irregular urothelial cell nests with bland cytology (on high‐power view), not involving the muscularis propria and adventitia
Pathological tumor stage	pT1, tumor invades subepithelial connective tissue (lamina propria)
Lymphovascular invasion	No lymphovascular invasion was noted
Concomitant CIS	Not detected

Abbreviation: CIS: carcinoma in situ.

### 2.3 Outcome and Follow‐Up

Despite offering the surgical treatment as the first‐line treatment and explaining the positive aspects of the curative procedure, the patient refused to undergo surgery and requested to be discharged from the hospital. After evaluating the capacity and competency of the patient to make a medical decision, he was discharged from the hospital according to his preference on 4th October 2023. He was convinced of the importance of the surgery. The hernia was manually relocated in the abdominal and pelvic cavity before the TURP, and histologic examination after that confirmed the diagnosis of urethral carcinoma. The patient's treatment continued as an outpatient, and during the next 6 months after the surgery, no specific events, recurrence, or relapse of the disease were seen (3rd April 2023). A whole‐body CT scan showed no metastasis or involvement of adjacent organs.

## 3 Discussion

Several studies have shown various prevalences of bladder observed in the hernia, ranging from 1% to 4%. One study evaluated the presence of bladder in hernia in 6361 cases of people over 50 and reached a result of 0.03% [13, 20]. Bladder hernias are categorized as (1) Paraperitoneal, where the peritoneum covers the external aspect of the herniated bladder; (2) extraperitoneal, small tumors that do not cover the peritoneum; and (3) intraperitoneal, where the entire portion of the herniated bladder is covered by the peritoneum [21]. Most bladder hernias are asymptomatic until their progression to a level that causes two‐step micturition (known as the Mery sign). The second step occurs due to pressure on the herniated part in the scrotum or inguinal area [22]. Less common manifestations are vesicoureteral reflux, renal dysfunction, and lithiasis [14, 23]. The incidence of intra‐inguinal hernia cancer is very low, seen in less than 1% of herniorrhaphies [14]. Most of these cancerous tumors are due to metastasis from other parts of the body, most commonly from the gastrointestinal system, with less than 10% of these cases originating from UCs [24].

The most common type of bladder malignancy is UC, which has a wide range of presentations, from asymptomatic good prognosis low‐grade UC, characterized by a low risk of progression, to high‐grade UC. High‐grade UC is categorized as non‐muscle‐invasive, which has a better prognosis than the latter but has the potential to progress to the muscle‐invasive form and involve adjacent parts. The muscle‐invasive form has the poorest prognosis and invades the bladder muscle and extra‐bladder structures [25]. The diagnosis of UC might be performed with various diagnostic tools such as ultrasound, CT urethrogram, and magnetic resonance imaging (MRI), as well as PET scans [4]. Recent advances in artificial intelligence and improvements in the sensitivity and specificity regarding these methods have considerably improved diagnostic accuracy [26].

The management plan also depends on the type of cancer and the degree of its progression (stage). Most low‐grade types are managed with conservative management and progression monitoring. However, those with more invasive histology and involvement of the bladder wall, muscle, or visceral fat are planned to be managed with surgical intervention, single or combination chemotherapy, immunotherapy, and advanced therapeutic regimens [27, 28]. Other factors should be considered for the optimal management of these cancers by predicting the risk of recurrence and progression of the malignancy. A higher number of lesions, larger size, previous history of disease recurrence, presence of carcinoma in situ, and higher grade and stage of the tumor are associated with a higher risk of recurrence and malignancy progression [27, 29, 30]. Previous studies have also identified genes that can play an essential role in BC's emergence, progression, and recurrence. The overexpression of some genetic factors, such as tumor suppressor genes (TP53), cell cycle regulatory proteins (ki‐67, HER 2, EGFR), extracellular and cell‐surface markers (E‐cadherin), chemotherapy response modifiers (ABCB 1 and ERCC 1), apoptosis regulators (BCL 2), and angiogenic factors (VEGF and PTGS 2), has shown to be correlated with a poorer prognosis. At the same time, other genetic factors play an opposite role [31, 32].

The diagnosis of UC of the bladder mainly depends on imaging modalities, especially CT scans and MRIs. There are controversies about the efficacy of PET scans for detecting perioperative lymph nodes and staging the disease. Cytology of the urine and cystoscopy are helpful means for screening and confirming UC [33, 34]. Technological advancements have improved the sensitivity and specificity in CT scans to diagnose bladder cancer [35]. Urine cytology has shown considerable specificity for diagnosing UC [36], and significant advancements have been made in the therapeutic methods for treating UC. More conventional strategies, such as transurethral resection of bladder tumor (TURBT), intravesical BCG therapies, and cystectomies, have been replaced by medications designed to target the cellular and molecular mechanisms underlying the immune response in UBC. The introduction of medications that affect immune checkpoints, such as PD‐1/PD‐L1 checkpoint blockade, has improved the survival of patients with bladder cancer and lowered the adverse effects that patients had to tolerate with conventional methods [37, 38]. Although bladder cancers with muscle invasion are primarily treated with surgical methods, intravesical therapeutic combinations, such as BCG plus immunotherapies, combined immunotherapies, and combined intravesical chemotherapies, are novel, effective treatments that can be used in less invasive cases [39]. However, these options could not be considered for our patient due to the invasion of the bladder muscles and their ineffectiveness [39].

Noteworthy, the presentation and diagnosis of bladder cancer, including UC, are not always straightforward. Approximately 1%–4% of inguinal hernias contain the bladder [40]. Some risk factors have been identified for the herniation of the bladder through the inguinal canal, such as benign prostatic hypertrophy, bladder underactivity, weak abdominal fascia, obesity, male gender, urinary outlet obstruction, and advanced age [40]. About 12% of inguinal hernias are reported to be associated with urinary tract malignancies (seven cases are mentioned in Table 3). Most of these cancers are asymptomatic until the advanced stage [40]. However, they can be manifested as bulging of the inguinal canal and permanency with lower volume after urination. There are several treatment options for these tumors, such as TURP after the reduction of the hernia when it is feasible, laparoscopic surgeries, and total or partial cystectomy in case of bladder wall (muscle) involvement [40, 42, 43].

**TABLE 3:** Bladder carcinomas detected in the inguinal, scrotal, or abdominal hernias.

Article and authors	Age and gender	Presentation	Treatment/progression or remission of the disease
A rare case of muscle‐invasive bladder cancer in a vesical inguinal hernia, Falabella et al. [14]	81‐year‐old male	The patient presented with macroscopic hematuria. Flexible cystoscopy and urinary cytology were inconclusive. A bladder inguinal hernia with diffuse thickening of the bladder wall was seen on total body CT	The inguinal hernia was reduced surgically, and a partial cystectomy was conducted to remove the suspicious cancerous part of the bladder. Muscle‐invasive squamous cell carcinoma was seen in histology—uneventful follow‐up for the next 2 years
Direct inguinal hernia containing bladder carcinoma: A case report and review of the literature, Katsourakis et al. [41]	79‐year‐old male	History of progressive left groin bulging enlarging for 10 years and TUR‐BT 3 months before because of papillary urothelial carcinoma of the urethra. A left groin hernia with an irregular mass growing out from the urinary bladder was detected on a CT scan	Partial cystectomy and repair of the inguinal hernia were performed through an inguinal approach. Histology showed urothelial carcinoma with invasion of 1/3 (T2a). Uneventful 1‐year follow‐up
Bladder cancer in an inguinoscrotal vesical hernia, Regis et al. [42]	79‐year‐old male	Presented with hematuria. A bladder dome tumor was seen in cystoscopy—unsuccessful transurethral resection. CT showed inguinoscrotal hernia with vesical carcinoma	Open surgical treatment, in addition to the repair of the hernia, was done. A high‐grade urothelial carcinoma (stage pT2b) with a free resection margin of < 1 mm was detected in histology. Radiotherapy was planned as an adjuvant long‐term treatment
Urothelial carcinoma of the urinary bladder related to a migrated mesh after inguinal hernioplasty: A case report, Sasaki et al. [43]	70‐year‐old woman	Had surgery for left inguinal hernia 5 years before the presentation with gross hematuria. Cystoscopy and computed tomography revealed a stone formed in the mesh, and histologic examination after partial cystectomy demonstrated muscle‐invasive bladder cancer. The patient has received adjuvant chemotherapy. High‐grade urothelial carcinoma, stage pT3a with positive lymph nodes, stage pN3, was the diagnosis	After the diagnosis, mesh removal surgery and partial cystectomy were done, and considering the histologic examination result, neoadjuvant chemotherapy and radical cystectomy were conducted. The pathological diagnosis was uneventful outpatient 3‐months of adjuvant chemotherapy recorded after the surgery
Vesical tumor within an inguinal bladder hernia: A case report, Binjawhar et al. [40]	65‐year‐old male	The patient presented with gross hematuria and right inguinal swelling. A preoperative CT scan detected a bladder neoplasm within a right inguinal vesical hernia. Invasive subepithelial connective tissue, suggestive of a high‐grade urothelial carcinoma, was diagnosed in postoperative histopathology	Underwent TURBT and hernia repair. Uneventful 2‐week outpatient follow‐up
Bladder cancer within a direct inguinal hernia: CT demonstration, Caterino et al. [44]	70‐year‐old	Presented with intermittent episodes of hematuria during cystitis. The ultrasonographic evaluation revealed a dysmorphic bladder with slight thickening of the right anterolateral bladder wall. High‐grade urothelial carcinoma was detected in the histologic examination. A dysmorphic bladder shifted to the right, and a bilateral inguinal hernia could be seen on a CT scan. The diagnosis was confirmed by cystoscopy	A voluminous solid neoplasm infiltrating the wall of the distal half of the diverticulum was found. Moreover, the right inguinal plastic with a Praline net was performed. The final diagnosis was bladder papillary carcinoma G2 T2
Bladder cancer in an inguinal vesical hernia Kandemirli et al. [15]	70‐year‐old male	Presented with intermittent right‐sided scrotal swelling for the last 4 months. In ultrasonographic evaluation, multiple papillary hyperechoic lesions with internal vascularity on Doppler ultrasound were protruding into the fluid‐filled cavity. CT showed the protrusion of the bladder through the inguinal canal into the scrotum	Urothelial carcinoma with invasion into the muscular layer was observed during hernioplasty, followed by TURP

To our knowledge, this is the first case of a bladder tumor found in the visceral hernia that has been reported. There are case reports regarding the detection of BC in the scrotal and inguinal hernia fields [8, 14]. However, the challenging diagnosis and the probable delay in detection in these cases make us pay more attention to this coincidence. Potentially delayed diagnoses are accompanied by a worse prognosis and require more invasive treatments [45]. Therefore, a more detailed and holistic evaluation of this coincidence seems necessary in future research.

## 4 Clinical Key Message (Conclusion)

Bladder cancer can be obscured in an inguinal or abdominal wall hernia and be roughly diagnosed with imaging, such as ultrasonography. Any symptom suggesting a bladder tumor, such as abdominal pain or fullness, should be considered a probable case of malignancy. Therefore, decreasing the risk of misdiagnosis by conducting appropriate imaging modalities is essential. The therapeutic approach depends on the tumor's location, the reducibility of the hernia, and the tumor's grade and stage.

## Author Contributions

P.E. conceptualization; data curation; formal analysis; project administration; supervision, methodology; supervision; visualization; writing – original draft; writing – review and editing. A.M. conceptualization; data curation; resources; software; validation. M.M. investigation; methodology; resources; validation; writing – original draft; writing – review and editing. D.J.G. writing – original draft; review and editing. M.T. data curation and verification (patient's history, computed tomography scan figures). F.M. data curation (pathologic features) and analysis of the pathologic findings.

## Consent

Written informed consent was obtained from the patient to publish this report following the journal's patient consent policy.

## Conflicts of Interest

The authors declare no conflicts of interest.

## Data Availability

The data that support the findings of this study are available on request from the corresponding author. The data are not publicly available due to privacy or ethical restrictions.
